# Women’s experiences of fear of childbirth: a metasynthesis of qualitative studies

**DOI:** 10.1080/17482631.2019.1704484

**Published:** 2019-12-20

**Authors:** Helena Wigert, Christina Nilsson, Anna Dencker, Cecily Begley, Elisabeth Jangsten, Carina Sparud-Lundin, Margareta Mollberg, Harshida Patel

**Affiliations:** a Institute of Health and Care Sciences, The Sahlgrenska Academy at University of Gothenburg, Gothenburg, Sweden; b Division of Neonatology, Sahlgrenska University Hospital, Gothenburg, Sweden; c Faculty of Caring Science, Work Life and Social Welfare, University of Borås, Borås, Sweden; d Chair of Nursing and Midwifery, School of Nursing and Midwifery, Trinity College Dublin, Dublin, Ireland

**Keywords:** Fear after childbirth, fear of childbirth, meta-synthesis, requests for caesarean section, women experience

## Abstract

**Purpose**: Women’s experiences of pregnancy, labour and birth are for some pregnant women negative and they develop a fear of childbirth, which can have consequences for their wellbeing and health. The aim was to synthesize qualitative literature to deepen the understanding of women’s experiences of fear of childbirth.

**Methods**: A systematic literature search and a meta-synthesis that included 14 qualitative papers.

**Results**: The main results demonstrate a deepened understanding of women’s experiences of fear of childbirth interpreted through the metaphor “being at a point of no return”. Being at this point meant that the women thought there was no turning back from their situation, further described in the three themes: To suffer consequences from traumatic births, To lack warranty and understanding, and To face the fear.

**Conclusions**: Women with fear of childbirth are need of support that can meet their existential issues about being at this point of no return, allowing them to express and integrate their feelings, experiences and expectations during pregnancy, childbirth and after birth.

Women with fear after birth, i.e., after an earlier negative birth experience, need support that enables them to regain trust in maternity care professionals and their willingness to provide them with good care that offers the support that individual women require. Women pregnant for the first time require similar support to reassure them that other’s experiences will not happen to them.

## Background

Women’s experiences of pregnancy, labour and birth are multidimensional (Larkin, Begley, & Devane, 2009) and can include all kinds of feelings, from joy and fulfilment to anxiety and horror. For some pregnant women, negative feelings take over and they may develop a fear of childbirth, which can have consequences for their wellbeing and health (Nieminen et al., 2017). Women’s fear of labour and birth is an important reason for the increasing number of requests for and rates of caesarean section (CS) in Europe, Australia and the USA (D’Alton & Hehir, 2015; OECD, 2017). The concept of fear of childbirth is broad, with no exact definition of the condition (Nilsson et al., 2018; O’Connell, Leahy-Warren, Khashan, Kenny, & O’Neill, 2017). There is lack of conformity in the tools used for measuring it (Richens, Lavender, & Smith, 2018), which, together with cultural differences, leads to variation in the global prevalence of women’s fear of childbirth.

When measured in the same way, the prevalence of intense fear of childbirth (FOC) varies from 4.8% in Australia (Toohill, Fenwick, Gamble, & Creedy, 2014) to 6.3% in Belgium and 14.8% in Sweden (Nilsson et al., 2018). Women’s feelings of uncertainty around the birthing process seem to be a basis for their fear of giving birth (Sheen & Slade, 2018). Fear arising before a woman’s first childbirth is labelled primary fear of childbirth, while secondary fear is often related to her earlier birth experiences (Hofberg & Ward, 2003; Lukasse, Schei, & Ryding, 2014; Størksen, Garthus-Niegel, Vangen, & Eberhard-Gran, 2013). Results from a recent systematic review on causes and outcomes of FOC demonstrate that a previous negative or traumatic birth experience and operative birth are the strongest predictors (Dencker et al., 2019), and consequently, suggested it was important to label fear caused by birth as ´fear after birth´ (FAB).

Although agreement on the best treatment for women with fear of childbirth has not yet been achieved, the available evidence demonstrates promising effects of some treatments on women’s fear and their experiences around birth. The number of randomized controlled trials (RCT) conducted on different prenatal interventions for fear of childbirth (Nieminen et al., 2017; Stoll, Swift Emma, Fairbrother, Nethery, & Janssen, 2018) is limited. However, some interventions have confirmed positive effects on women’s fear (Stoll et al., 2018) as different kinds of counselling focused on women’s fear and previous distressing birth experiences (Gamble, Toohill, Creedy, & Fenwick, 2015; Saisto, Salmela-Aro, Nurmi, Könönen, & Halmesmäki, 2001; Toohill et al., 2014), prenatal childbirth education (Haapio, Kaunonen, Arffman, & Åstedt-Kurki, 2017; Serçekuş & Başkale, 2016), and yoga (Newham, Wittkowski, Hurley, Aplin, & Westwood, 2014). Moreover, other benefits of interventions for women are a reduction in overall CS rates, and vaginal birth as a more frequent first choice in their following pregnancy (Fenwick, Toohill, Creedy, Smith, & Gamble, 2015). Women also describe midwife-led counselling as increasing their confidence in giving birth, which made them feel safer, with a positive influence on their birth experience (Larsson, Hildingsson, Ternström, Rubertsson, & Karlström, 2019; Larsson, Karlström, Rubertsson, & Hildingsson, 2015; Ryding, Persson, Onell, & Kvist, 2003).

Women’s experiences of fear of childbirth seem to be related to their emotional well-being, stress symptoms, impact on everyday life, and wishes for a CS on their next birth (Klabbers, Javbh, Mavdh, & Vingerhoets, 2016). Women fearing childbirth can feel a lack of confidence in birth, being influenced by negative birth stories, fear labour pain or losing control, and fear physical injury during birth. Women giving birth again after a previous negative birth experience often fear a repeated poor birth experience (Fenwick et al., 2015; Klabbers et al., 2016; Sheen & Slade, 2018). Fear of childbirth is a multifaceted condition (Dencker et al., 2019) and women with fearing childbirth are a heterogeneous group (Klabbers et al., 2016; Rondung, Ekdahl, Hildingsson, Rubertsson, & Sundin, 2018). To our knowledge, there is no metasynthesis conducted on women’s experiences of fear of childbirth. Such study is needed to deepen our knowledge of women’s experiences, which can be used to develop woman-focused interventions for fear of childbirth. The aim was to synthesize published, qualitative literature to deepen the understanding of women’s experiences of fear of childbirth.

## Methods

### Design

The current review describes a meta-synthesis based on the interpretative meta-ethnography method described by Noblit and Hare (Noblit & Hare, 1988). Meta-synthesis attempts to integrate results from a number of different but inter-related qualitative studies. The technique has an interpretive, rather than aggregating intent (Walsh & Downe, 2005) with emphasis on careful interpretation from the research included in the review.

### Search and selection strategy

A search strategy was developed, and reviewed for accuracy by one member not involved in its development (CS-L), using the Peer Review of Electronic Search Strategies (PRESS) criteria (CADTH Methods and Guidelines, 2016). No restrictions were applied to years searched, but papers included were limited to English and Swedish publications only. We searched electronic bibliographic databases of The Cochrane Library, PubMed, Scopus, PsycINFO and CINAHL from their inception dates in March 2015. An updated search was done in April 2018. Search strategy is described in Additional file 1.

#### Inclusion criteria

For inclusion criteria, we used the PICO tool for an effective search strategy to find qualitative studies (Methley, Campbell, Chew-Graham, Mcnally, & Cheraghi-Sohi, 2014). Problem: Fear of childbirth. Interest: Women’s experiences and views. Context: Childbearing women (defined as the period covering pregnancy, labour and birth, and 5-years postpartum).

*Selection of studies* Studies were selected for inclusion from the papers identified by team members working in pairs, using the above criteria. Any disagreements were resolved by a third member.

#### Results of search and selection strategy

For the whole research project, including two systematic reviews (Dencker et al., 2019; Nilsson et al., 2018), in total, 19,410 citations were identified. After removing duplicates 13,125 unique citations were screened by title and abstract and 12,623 excluded. Reasons for exclusion were: (a) duplicates, (b) topic not relevant to fear of childbirth, (c) studies explored the experiences of family, partner or health-care personnel (d) review articles, dissertations, quantitative and mixed-method studies. Full-text papers of the remaining 502 citations were assessed for eligibility and 362 of these were excluded. One hundred and forty full-text papers were screened for inclusion and of these 62 papers were excluded due to quantitative design. Due to the wealth of published research, we did not undertake additional searches of the grey literature.

**Figure 1.**
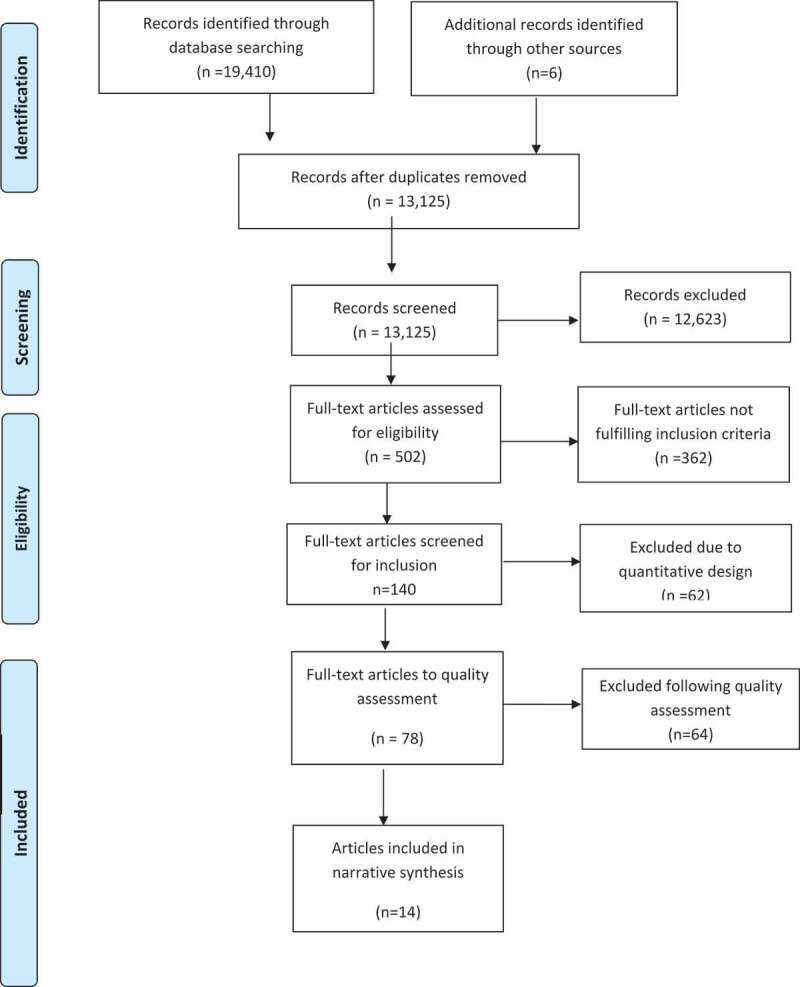
Flow Diagram

### Quality assessment and data extraction of included studies

The quality of the remaining 78 qualitative articles meeting the inclusion criteria for the quality assessment was assessed using the COREQ criteria (Tong, Sainsbury, & Craig, 2007) an accepted rating 32-item checklist tool for qualitative research. However, ethical issues, thorough use of the literature, quality and audit mechanisms, relevance and transferability were not included in the COREQ tool. Thus, 13 other items were incorporated into the checklist (items 9,10,12,13,33–35,40–45) and four items were adapted (items 8,26,28,29) (Table I), based on the work of Walsh and Downe (Walsh & Downe, 2006) and Lundgren et al. (Lundgren, Begley, Gross, & Bondas, 2012). This composite grid was useful in determining the quality of the papers and assisting the decision for inclusion or exclusion. The quality was rated as high if 39–45 marks were awarded, medium if 31–38 and low if ≤30. Each reviewer pair made quality assessments individually and then compared with others in the group for agreement. Ambiguous results or disagreements were discussed until consensus was reached. One of the team-members was a co-author on some of the articles (CN); others evaluated those in the reviewer team. Following quality assessment 64 papers were rated as having a low quality (score ≤ 30), and therefore excluded (Figure 1 and Table I), leaving 14 articles included in our synthesis (Figure 1). Using a pre-designed data extraction form, data on women’s experiences of fear of childbirth were extracted independently by each member of the four review teams and checked for accuracy by the paired reviewer.

**Table I.** Characteristics of included studies and quality assessment

Reference, Author, year, country	Study aims	Methodology and theoretical perspective	Sample	Quality assessment
Fenwick, Staff, Gamble, Creedy, & Bayes, (2010) Australia	To describe Australian women’s request for CS in the absence of medical indicators in their first pregnancy	Interviews Thematic analysis	8 primiparous and 6 multiparous women who requested CS without any known medical indication, all had experienced CS within the past 5 years	M:35
Fisher, Hauck, & Fenwick, (2006) Australia	To explore in detail the childbirth experiences of women identified as fearful of birth	Interviews Constant comparison	8 primiparous and 14 multiparous women who expressed fear, 19 woman were interviewed after childbirth and 3 women before	M:32
Melender, (2002) Finland	To describe the causes of fear associated with preg-nancy and childbirth and to describe coping stra-tegies of preg-nant woman who have fears	Interviews Content analysis	10 primiparous and 10 multiparous women interviewed 2–3 days after childbirth	M:31
Faisal, Matinnia, Hejar, & Khodakarami, (2014) Iran	To gain a deeper understanding of why Iranian primigravidae request CS without any medical indication	Interviews Thematic analysis	14 nulliparous women who requested CS without any medical indication, interviewed in the third trimester	M:38
Lyberg & Severinsson, (2010) Norway	To illuminate mothers’ fear of childbirth and their experiences of the team-midwifery care model during pregnancy, child-birth and the postnatal period	Interviews Hermeneutical analysis	4 primiparous and 9 multiparous women interviewed 1–1,5 year after childbirth	M:33
Ramvi & Tangerud, (2011) Norway	To investigate women requesting CS, but gave vaginal birth in spite of fear of childbirth	Interview Narrative approach	2 primiparous and 3 multiparous women interviewed 1 year after the childbirth	M:35
Eriksson, Jansson, & Hamberg, (2006) Sweden	To investigate and describe how intense fear related to childbirth is experienced, dealt with and communicated from the perspective of the women themselves	Interviews Grounded theory	6 primiparous and 14 multiparous women with experiences of intense fear, interviewed after childbirth	M:36
Nilsson, Bondas, & Lundgren, (2010) Sweden	To describe the meaning of previous experiences of childbirth in pregnant woman who have exhibited intense fear of childbirth such that it has an impact on their daily lives	Interviews Phenomeno-logical analysis	9 pregnant multiparous women who sought help for fear of childbirth	M:32
Nilsson & Lundgren, (2009) Sweden	To describe woman’s lived experience of fear of childbirth.	Interviews Phenomeno-logical analysis	2 pregnant primiparous and 6 pregnant multiparous women who sought help for fear	M:33
Nilsson, Robertson, & Lundgren, (2012) Sweden	To describe the meaning of fear of childbirth and of birth in women who earlier had experienced intense fear of childbirth after a previous negative childbirth experience	Interviews Phenomeno-logical analysis	6 multiparous women who sought help for fear of childbirth about 10 years earlier	M:34
Ryding, Wijma, & Wijma, (1998) Sweden	To describe women’s thoughts and feelings during the delivery process ending in ECS.	Interviews Phenomeno-logical analysis	29 primiparous and 24 multiparous women interviewed 1–5 days after elective CS	M:37
Salomonsson, Bertero, & Alehagen, (2013) Sweden	To apply and test the concept of self-efficacy to expectations of the up-coming birth in the context of severe fear of childbirth	Interviews Content analysis	17 pregnant primiparous women with fear at 25–26 gestational week, interviews between 32–38 gestation week	M:31
Wahlbeck, Kvist, & Landgren, (2018) Sweden	To describe women’s experience of undergoing art therapy with severe fear of childbirth.	Interviews Phenomeno-logical hermeneutical analysis	19 women who had undergone art therapy for severe fear of childbirth 10 multiparous and 10 primiparous, interviews 3 months after childbirth	M:36
Roosevelt & Kane Low, (2016) USA	To explore woman’s experiences while completing the W-DEQ, an instrument used to measure fear of childbirth.	Focus groups Interviews Content analysis	22 women who were pregnant or had given birth in the last 5 years, have self-identified fear of childbirth	M:31

Assessment for quality using the COREQ 32-items checklist, M = Moderate quality, 31–38 scores.

### Analysis and synthesis of included studies

The selected studies were distributed among four pairs of reviewers to read several times and for the extraction of detailed data. The papers’ results were then re-read and preliminary themes and concepts were extracted by four of the authors (HW, CN, EJ, HP) from the descriptions. Further themes and concepts were identified, compared and synthesized with the previous themes and concepts, whilst maintaining an open mind and returning to the initial themes and concepts to ensure reciprocal translation (Noblit & Hare, 1988). In a comparative process between the meta-synthesis and the detail of the individual study findings, themes emerged that were modified to encompass all data. The final stage of analysis involved grouping and integrating the key concepts to express these explanations in a main theme (Noblit & Hare, 1988). Reliability checks were undertaken by four reviewers (HW, CN, EJ, HP) on the derived themes and key concepts, resulting in a high level of agreement (Table II).

**Table II.** Main theme, themes and subthemes

Main theme	Being at a point of no return	
**Themes**	**Subthemes**	Articles
To suffer consequences from traumatic births	To live with terrible birth experiences	35,36,38,39,41–44,46,47
	To live with stories of terrible childbirth	34–37,39–42,46
To lack warranty and understanding	To lose control	34–44,42,45–47
	To lack understanding	35,39,40,42,46,47
	To fear pain and injury	34–37,39,41–44 46,47
To face the fear	To manage the fear	35,36,40,42 45,46
	To seek support	34,35,38–40,43,45,46
	To have caesarean section as an emergency exit	34,37–39,44

Studies originated from Australia (Fenwick et al., 2010; Fisher et al., 2006), Finland (Melender, 2002), Iran (Faisal et al., 2014), Norway (Lyberg & Severinsson, 2010; Ramvi & Tangerud, 2011), Sweden (Eriksson et al., 2006; Nilsson et al., 2010; Nilsson & Lundgren, 2009; Nilsson et al., 2012; Ryding et al., 1998; Salomonsson et al., 2013; Wahlbeck et al., 2018), and the USA (Roosevelt & Kane Low, 2016). Interviews with women were performed during pregnancy (Faisal et al., 2014; Fisher et al., 2006; Nilsson et al., 2010; Nilsson & Lundgren, 2009; Roosevelt & Kane Low, 2016; Salomonsson et al., 2013) and after birth (Eriksson et al., 2006; Fenwick et al., 2010; Lyberg & Severinsson, 2010; Melender, 2002; Nilsson et al., 2012; Ramvi & Tangerud, 2011; Roosevelt & Kane Low, 2016; Ryding et al., 1998; Wahlbeck et al., 2018). The women’s experiences were requested concerning different aspects of the following phenomena related to fear of childbirth: experiences (Eriksson et al., 2006; Nilsson & Lundgren, 2009; Roosevelt & Kane Low, 2016), dealing with and communication of fear (Eriksson et al., 2006), requesting CS without medical indication (Faisal et al., 2014; Fenwick et al., 2010; Ramvi & Tangerud, 2011), women’s childbirth experiences (Fisher et al., 2006; Lyberg & Severinsson, 2010; Nilsson et al., 2010; Ryding et al., 1998), long-term perspectives (Nilsson et al., 2012), experiences of the team-midwifery care model (Lyberg & Severinsson, 2010), experience of art therapy for fear (Wahlbeck et al., 2018), causes (Melender, 2002), coping strategies to deal with fear (Faisal et al., 2014), and self-efficacy concept related to the fear (Salomonsson et al., 2013).

## Results

The women’s experiences of fear of childbirth are interpreted through the metaphor “being at a point of no return”. Being at this point meant that the women thought there was no turning back from their situation. The women suffered consequences from traumatic birth experiences, thoughts of previous childbirth or from hearing other women told about their terrible experience of childbirth. The women were in a circumstance where they had no guarantee of a successful birth, which entailed feelings of losing control and fearing birth pain and birth injuries to themselves and their baby. Being at this point also involved meeting a lack of understanding of their situation from others, and not recognizing themselves in feelings and reactions. Their experiences of no return forced the women to face the fear eventually, either by developing different strategies to handle their fears, seeking support or, like an emergency exit, requesting to have a CS. This interpretation is described in detail in the following three themes and eight subthemes.

### To suffer consequences from traumatic births

#### To live with terrible birth experiences

The majority of multiparous women did not feel any fear during the first pregnancy, but after having experienced a terrible birth they were terrified about another experience of traumatic childbirth (Fisher et al., 2006; Lyberg & Severinsson, 2010; Melender, 2002; Nilsson et al., 2010; Nilsson & Lundgren, 2009; Nilsson et al., 2012; Ramvi & Tangerud, 2011; Ryding et al., 1998). The women stated that they had strength and trust in their own body before their first labour and birth (Nilsson et al., 2010) and questioned themselves as to why they could not give birth to a child like other women (Fisher et al., 2006). The time between the two pregnancies was distressing, since they knew what was awaiting them during the coming childbirth and feared that the same scenario would be repeated (Nilsson et al., 2012).

*I’d had a traumatic birth with my son and I was frightened that that was going to be repeated and as it was coming closer to my daughter (second child) being born, the fear increased and increased and I was petrified. I really thought that the same sort of things (sic) were going to happen* (Fisher et al., 2006), p. 71.

The traumatic birth experience included both trauma for the woman and fears for the health of the child (Nilsson et al., 2010). The painful experience of childbirth (Nilsson & Lundgren, 2009; Ryding et al., 1998), either a short (Fisher et al., 2006) or pro-longed labour (Fenwick et al., 2010; Fisher et al., 2006; Ryding et al., 1998) was experienced as being in a “torture chamber” (Nilsson et al., 2012). Some women received emergency CS due to threatened asphyxia of the baby (Ryding et al., 1998), and vacuum extraction that was associated with fear of their child’s death (Ramvi & Tangerud, 2011). Some of the women had experienced a stillborn child (Ramvi & Tangerud, 2011; Ryding et al., 1998) or injuries during the birth process (Ramvi & Tangerud, 2011).

Some of the women described similar “near-death” experiences, in which they became apathetic during the birth. Their bodies gave up, with the feeling of falling into a big black hole (Nilsson et al., 2010; Nilsson & Lundgren, 2009). They concentrated on surviving childbirth (Nilsson et al., 2010) but seemed to lose control of what was happening to their body, with a feeling of being outside of their body at times (Nilsson et al., 2010; Ramvi & Tangerud, 2011; Wahlbeck et al., 2018). The women tried to communicate with the midwife about their feelings (Nilsson et al., 2010, 2012) but experienced that they were left alone, abandoned with feelings of powerlessness (Nilsson et al., 2010; Roosevelt & Kane Low, 2016).

*It was a sort of a long black hole. It felt like no letup, just endless, endless pain. Nothing to hold on to … . The feeling in my chest of becoming totally empty … . It’s that feeling … drained of strength and energy and zest for life* (Nilsson et al., 2010), p. 304.

Some of the women described that they were bearing a deep sorrow from a previous traumatic birth experience (Nilsson et al., 2010, 2012; Ramvi & Tangerud, 2011). Some women, who had given birth before, felt bitterness towards the midwives who they felt had destroyed their lives (Ramvi & Tangerud, 2011). During the first childbirth, the women felt that they were not involved in the childbirth process (Fisher et al., 2006; Lyberg & Severinsson, 2010; Nilsson et al., 2010). It was the health-care professionals who were in command and the focus was on medical techniques and routines for safe birth (Fisher et al., 2006; Nilsson et al., 2010). The women did not receive the needed support from midwives. Hence, some midwives were described as emotionless (Lyberg & Severinsson, 2010; Nilsson et al., 2010; Nilsson & Lundgren, 2009). Furthermore, the midwives did not respect women’s wishes to participate in their own childbirth and women sometimes met with an un-caring attitude (Nilsson et al., 2010; Nilsson & Lundgren, 2009).

*And she (midwife) was very cold and very hard and didn’t touch me in any way. She was sitting on the floor looking at me, she sort of couldn’t, she didn’t talk to me properly and wasn’t a gentle kind of person. I sort of felt that I couldn’t choose myself; I wanted somebody to guide me … . I didn’t understand anything about all that, thought it was really weird … it was as if she didn’t believe that I was in pain* (Nilsson et al., 2010), p. 302.

For women, a difficult postpartum period followed after the traumatic childbirth experience. Women reported that they experienced chaos; pain, difficulty sleeping and nightmares about childbirth (Ramvi & Tangerud, 2011). Strong feelings of fear of childbirth led women to think twice before getting pregnant again and some chose to have fewer children than they really desired (Nilsson et al., 2012).

*Well, it felt more like an action of the will that I’m putting the fear aside because now I want another child. But that also took 4 years. I think I needed those years to get the strength to think about it again that I would go through this again* (Nilsson et al., 2012), p. 262.

#### To live with stories of terrible childbirth

Women described how they heard other women, mothers and sisters, tell about their traumatic experience of childbirth (Faisal et al., 2014; Fenwick et al., 2010; Fisher et al., 2006; Melender, 2002; Ramvi & Tangerud, 2011), and the thought of bodily injuries after childbirth scared the women (Faisal et al., 2014).

*When I was born I almost killed my mother. It was a twenty-four hour labour and she had two hundred and seventy internal stitches* (Fenwick et al., 2010), p. 397.

First time mothers had seen films showing how women may die during childbirth, seen pictures or read books about horrific births (Faisal et al., 2014; Fisher et al., 2006; Melender, 2002) and, lacking their own experience of giving birth, they were influenced by other people’s lived experiences (Faisal et al., 2014). Even what the women saw of birth in the mainstream media was terrifying (Roosevelt & Kane Low, 2016).

*Well I think the biggest thing is being a first time mum, a lot of people choose to be brutally honest about childbirth and they tell you all the horror stories, the ones you don´t want to hear (*Fisher et al., 2006*), p. 69.*

The constant presence of fear in thoughts and body affected women both physically and mentally (Eriksson et al., 2006; Wahlbeck et al., 2018). They had poor appetite, were tense, had mood swings, difficulty with concentration, experienced stomach ache, poor sleep (Nilsson & Lundgren, 2009), or nightmares about childbirth (Eriksson et al., 2006; Ramvi & Tangerud, 2011). They were scared and had poor self-confidence and doubts about their own capacity to give birth to a child (Nilsson et al., 2010; Wahlbeck et al., 2018).

*I remember waking up in cold sweat terrified by the very thought of the forthcoming delivery* (Eriksson et al., 2006), p. 242.

### To lack warranty and understanding

#### To lose control

Some of the women had been scared since they were teenagers, while others felt fear first when they planned a pregnancy or became pregnant. Regardless of the timing for fear of childbirth, women were constantly thinking day and night about future childbirth (Eriksson et al., 2006). The ever-present fear was experienced as pure mental torture (Fenwick et al., 2010). The women described themselves as caught in their own body where there was no turning back, and they had to go through labour and birth (Lyberg & Severinsson, 2010; Nilsson & Lundgren, 2009), with no guarantee of having a successful birth (Melender, 2002; Nilsson & Lundgren, 2009; Roosevelt & Kane Low, 2016).

*I can feel at the moment the sense of being perhaps slightly entrapped, that I’m not getting out of this. I have to somehow choose something because the baby has to come out* (Nilsson & Lundgren, 2009), p. e5.

The nulliparous women described fear of the unknown, not knowing what would happen, as they had no previous experience of childbirth (Fisher et al., 2006; Melender, 2002). They did not have enough knowledge of what could happen during birth (Melender, 2002). Some multiparous women showed fatalism and described themselves as powerless. They believed that labour and birth was completely impossible to control and believed that fate controlled everything (Eriksson et al., 2006; Salomonsson et al., 2013). They thought they had had good luck earlier and asked themselves why would it be good this time (Fisher et al., 2006).

*I had had a pretty good experience last time (I gave birth) and can you win lotto twice or will this time be really horrendous with intervention and stuff like that? That’s my fear this time* (Fisher et al., 2006), p. 71.

Some women were afraid of losing control of themselves during labour and birth and screaming uncontrollably (Faisal et al., 2014; Fisher et al., 2006; Lyberg & Severinsson, 2010; Nilsson & Lundgren, 2009; Ramvi & Tangerud, 2011; Roosevelt & Kane Low, 2016; Wahlbeck et al., 2018). Multiparas described their vulnerability during childbirth, especially when being naked and with intimate areas visible (Lyberg & Severinsson, 2010). The women also feared their wishes not being fulfilled and their dignity not being respected by the professionals (Fisher et al., 2006; Roosevelt & Kane Low, 2016).

*I didn’t want to make too much noise. I wanted to be like one of those pretty ladies on TV who just pushes the baby out and everyone is happy and peaceful. Turns out I was really loud and I’m still embarrassed about that* (Roosevelt & Kane Low, 2016), p. 35.

#### To lack understanding

It was obvious that people surrounding these women did not understand their fear of childbirth. Some women felt that, because of their fear, they were not considered as “real” women as they could not cope normally (Nilsson & Lundgren, 2009; Ramvi & Tangerud, 2011). The discrepancy was noted between the woman’s own image of herself as a woman with strength and positive expectations and the impression of her implied by other people (Nilsson & Lundgren, 2009). They felt guilt and were ashamed of this (Nilsson & Lundgren, 2009; Ramvi & Tangerud, 2011).

*If I manage to give birth to a child vaginally, which is for me the natural or normal way, then I will really be a member of this club, i.e. the women’s club* (Nilsson & Lundgren, 2009), p. e6.

The women felt left out due to lack of understanding of their fear. It was difficult to talk about it (Fisher et al., 2006; Roosevelt & Kane Low, 2016; Wahlbeck et al., 2018) when they were seen as abnormal. They lacked support for their fear and were met by the attitude that women have given birth to children for many generations and it is just a natural thing to do (Eriksson et al., 2006; Fisher et al., 2006; Ramvi & Tangerud, 2011; Roosevelt & Kane Low, 2016). The women had to face lack of understanding from the professionals, and not being listened to, and were humiliated by them when they expressed their desire to have a CS due to fear (Eriksson et al., 2006; Ramvi & Tangerud, 2011).

*You get this feeling that you’re not as good as everyone else or that what you feel is not normal* (Eriksson et al., 2006), p. 244.

#### To fear pain and injuries

Some of the nulliparous women feared pain during childbirth. They had heard from other women about severe pain during childbirth, which they knew they would have to endure to survive (Fisher et al., 2006). Their worry about coping with pain sometimes made them ask for CS (Faisal et al., 2014).

*Labour pain is the most frightful thing. Thinking about labour pain is something that frightens me about vaginal delivery. What will I do if I had a lot of pain?* (Faisal et al., 2014), p. 229.

Women also described their fear of injuries to their body (Melender, 2002; Wahlbeck et al., 2018). Some women, who had exceeded their expected due date, had previously birthed a large child leading to severe perineal tears (Melender, 2002) and believed that vaginal birth was associated with physical injuries (Fenwick et al., 2010). On the other side, women who had planned CS or emergency CS earlier, feared for both physical and mental injuries (Fisher et al., 2006; Ryding et al., 1998). The women also expressed concern that their babies might be injured physically or would have a malformation (Faisal et al., 2014; Fenwick et al., 2010; Fisher et al., 2006; Melender, 2002; Roosevelt & Kane Low, 2016; Ryding et al., 1998; Wahlbeck et al., 2018), or might die during labour and birth (Fisher et al., 2006; Ryding et al., 1998; Wahlbeck et al., 2018).

*I want to do the best and everything that is necessary, I could make sure, I will have a healthy baby without any physical harm* (Faisal et al., 2014), p. 229.

Guilty feelings towards the baby were also expressed because of their fear (Fisher et al., 2006; Nilsson & Lundgren, 2009) as some believed that their fear might injure the baby in utero as though it was an unwanted child (Ramvi & Tangerud, 2011). Women were also worried about their successful future role as a good mother (Nilsson & Lundgren, 2009; Wahlbeck et al., 2018). The women’s suffering influenced their bond with the children leading to lack of loving expression to the baby (Nilsson et al., 2010; Ramvi & Tangerud, 2011) or, conversely, being over-protective (Nilsson et al., 2012).

*Children usually like to stand between the adult’s legs. Sort of creep in there. And that gives me a pounding heart and you know physical like this, shivers … . It felt like this is something that has to do with the childbirth. And it took many years. Now I’m noticing that it will soon be 13 years ago. It’s okay now* (Nilsson et al., 2012), p. 261.

### To face the fear

#### To manage the fear

The women used different strategies for managing their fear. They forced themselves to face their fear by focusing on, and trusting, their own ability for normal childbirth (Nilsson & Lundgren, 2009; Salomonsson et al., 2013; Wahlbeck et al., 2018). They tried to confront their memories to understand what had happened to regain confidence (Nilsson et al., 2012). They convinced themselves that women across the ages had managed childbirth and that they have to manage this too (Eriksson et al., 2006; Fisher et al., 2006; Melender, 2002; Nilsson & Lundgren, 2009; Salomonsson et al., 2013).

*I am not the first one to do this, so I try to think that I will manage like everyone else* (Salomonsson et al., 2013), p. 198.

Other strategies to cope with fear included blocking memories from previous childbirth experiences by avoiding talking to others about their fear (Nilsson & Lundgren, 2009), avoiding thoughts associated with childbirth and choosing to refrain from joining parenting groups. Women also distracted themselves by being busy with different activities, hoping that the fear would disappear when they concentrated on something else (Eriksson et al., 2006).

The women sought information about childbirth through books and professional support from maternity care. They received information on pain relief during visits to the antenatal clinic and the maternity care department (Eriksson et al., 2006; Melender, 2002). Managing the fear was also related to having tests showing the healthy child, and controlling the baby’s movement in utero (Melender, 2002). Women also used other coping strategies, e.g., writing a letter to the obstetrician and midwives about their wishes around their birth (Eriksson et al., 2006).

*I contacted the fear of childbirth team rather early because my attitude was to fight the fear so that I could deliver in the normal way, but it didn’t work, so it ended up being a cesarean section* (Eriksson et al., 2006), p. 246.

The women desired control over their own body, thinking that their body had the capacity to handle the fear and hoping to rely on their own resources. Furthermore, they felt that if they lost control over the situation in spite of their own self-efficacy, there would be people there who knew how to handle the situation (Salomonsson et al., 2013). It was understood by women that they had to prepare actively to confront and manage their childbirth fear. The women used relaxation and breathing techniques to relieve their fear and tried to work with their body instead of against it (Eriksson et al., 2006; Salomonsson et al., 2013).

*Yes, in one way I want to (leave the control to the body). In some way it will be easier because my stupid head will not be in the way. In another way I wish that I could take over the body* (Salomonsson et al., 2013), p. 197.

#### To seek support

The fear was relieved once women became involved in the birth process through receiving information and guidance from midwives about what was going to happen (Ramvi & Tangerud, 2011; Salomonsson et al., 2013). The fear also decreased when they felt they were listened to, confirmed, respected and could build up a good caring relationship with their midwives. Women who had a good conversation with the midwife experienced good support regarding fear of childbirth, which increased their self-esteem (Lyberg & Severinsson, 2010). A supportive midwife along with an understanding family and husband had healing effects on fear of childbirth experiences (Fisher et al., 2006; Ryding et al., 1998).

*I stopped worrying about the birth and thought this will be good, I trusted the midwife fully. She was there all the time during labour, even when the birth took a long time she was there with me. She guided me, she listened to me and I could concentrate on giving birth* (Lyberg & Severinsson, 2010), p. 387.

Women were dependent on the midwives’ competence and skills (Lyberg & Severinsson, 2010). The majority of the women had no control over the situation during childbirth and understood that it was important to believe in and trust the midwives. The majority of women had surrendered to the midwives because of uncertainty related to fear of childbirth (Fenwick et al., 2010). Giving authority to the skilled professionals was perceived as a relief as the responsibility was not there anymore (Salomonsson et al., 2013).

*and I thought that if things go wrong, although I wasn’t in control myself, I knew that were people around me to look after me … I trusted them. I handed control of myself over to them. I was completely in their hands* (Fenwick et al., 2010), p. 397–398.

Another strategy for managing a fear of childbirth was to focus on the future. Pregnant women were wondering about how it would be to meet their child and how their future life with the child would look (Salomonsson et al., 2013; Wahlbeck et al., 2018). Talking with other people about their concern and receiving support from friends and family relieved their fear (Eriksson et al., 2006; Melender, 2002; Salomonsson et al., 2013).

Women who received art therapy as a part of treatment for severe fear of childbirth during pregnancy were positive towards the therapy. By sharing and making the fear visible in the creation of images in paintings, the women were able to gain hope and self-confidence. The painting was an important tool to promote inner healing and they could then face their fear (Wahlbeck et al., 2018).

*Then it just came and it was so fantastic to let these emotions come up in the image—that it’s possible!! It made it easier to handle the fear and anxiety* (Wahlbeck et al., 2018), p. 5.

#### To have caesarean section as an emergency exit

Some women with a pronounced fear of childbirth wanted to be delivered by CS to feel safe, to have control over the childbirth, and to have a peaceful and perfect childbirth (Faisal et al., 2014; Fenwick et al., 2010; Ramvi & Tangerud, 2011). The women said that their grandmothers and friends had given birth through CS without any problems (Fenwick et al., 2010). Some of the nulliparous women were terrified by the thought of vaginal childbirth (Faisal et al., 2014) and they absolutely could not imagine a vaginal birth (Ramvi & Tangerud, 2011).

*I would have sacrificed the child in order not to give birth. The episode when I was told that I had to give birth was awful. I felt as if I was going to die and the doctor was so tough when he said it. I felt that he was angry* (Ramvi & Tangerud, 2011), p. 272.

Women indicated that fear of childbirth was a dominant factor to ask for CS (Ryding et al., 1998), as possible alternative for childbirth (Eriksson et al., 2006), and ignored the risks of CS, believing that all the options were associated with risk (Fenwick et al., 2010). Some women, although they talked to the obstetrician and a fear of childbirth team and asked for CS (Eriksson et al., 2006), did not actually want CS (Lyberg & Severinsson, 2010). On the contrary, some women described how their expression of the desire to have a CS was rejected and not being heard was perceived as an inhumane decision (Ramvi & Tangerud, 2011).

*I could not feel happy about my pregnancy because I was so anxious about giving birth. I did not really know why I was so concerned, the only thing I thought was that if I can have a caesarean section I won’t have to worry about it, but in my innermost mind I did not want a caesarean* (Lyberg & Severinsson, 2010), p. 386

## Discussion

This meta-synthesis brings a greater understanding of fear of childbirth, from the perspective of women, from the merged qualitative findings. The main results demonstrate a deepened understanding of women’s experiences of fear of childbirth interpreted as “*being at a point of no return”* with three main themes and eight subthemes.

Pregnancy and birth are not only physical events but also existential events loaded with deep and unique meanings for the woman. The main theme, “being at a point of no return”, can be related to existential meanings of giving birth. Larsson (Larsson, 2018) describes in her thesis existential and spiritual dimensions of childbearing, pregnancy and childbirth as “a mystery” that has to be reflected and integrated into the unique woman’s life as a meaningful experience. Childbirth is also described as a “border situation” that can entail both strength and suffering (Lundgren, 2018). The results of this meta-synthesis indicate that women who fear childbirth are in a severe existential situation in which they experience suffering, loneliness, lack guaranties and understanding, and experience a loss of control when having to face what they fear most; pregnancy, labour and birth. For the women who have had a negative birth experience, experiences of post-traumatic stress together with a lack of trust in maternity care and its providers (midwives and obstetricians) can have major significance for their fear (Dencker et al., 2019), and make their situation even more difficult. Individual existential meanings of pregnancy and birth may be difficult for women to verbalize in a technocratic maternity care in which childbirth often is acknowledged as a problem in the eyes of health professionals and organizations. Maternity care is not usually focused on addressing existential issues, instead it tends to objectify childbirth and respond to women’s problems with various medical techniques. For women with fear of childbirth, such care may lead them to experience a deeply felt loss of meaning in relation to pregnancy, labour and birth (Crowther & Hall, 2018; Larsson, 2018).

Most of the multiparous women in the reviewed studies described that fear of childbirth followed after a terrible birth, with great pain experienced, that women were afraid would be repeated (Fenwick et al., 2010; Fisher et al., 2006; Lyberg & Severinsson, 2010; Melender, 2002; Nilsson et al., 2010; Nilsson & Lundgren, 2009; Nilsson et al., 2012; Ramvi & Tangerud, 2011; Ryding et al., 1998). The women felt that their bodies gave up during the birth (Nilsson et al., 2010; Nilsson & Lundgren, 2009; Ramvi & Tangerud, 2011; Wahlbeck et al., 2018) and that the health-care professionals were in command (Fisher et al., 2006; Lyberg & Severinsson, 2010; Nilsson et al., 2010), yet they felt abandoned (Nilsson et al., 2010; Roosevelt & Kane Low, 2016), particularly by emotionless midwives (Lyberg & Severinsson, 2010; Nilsson et al., 2010; Nilsson & Lundgren, 2009), towards whom they sometimes felt bitter (Ramvi & Tangerud, 2011). Women lived with stories of terrible childbirth, they heard other women telling of their traumatic experiences of childbirth (Faisal et al., 2014; Fenwick et al., 2010; Fisher et al., 2006; Melender, 2002; Ramvi & Tangerud, 2011) or had seen films, or read books about horrific births (Faisal et al., 2014; Fisher et al., 2006; Melender, 2002). Women were met with misunderstanding, and they felt they were not considered as “real” women (Nilsson & Lundgren, 2009; Ramvi & Tangerud, 2011). They found it difficult to talk (Fisher et al., 2006; Roosevelt & Kane Low, 2016; Wahlbeck et al., 2018), lacked support and sympathy for their fear of childbirth and were met by the attitude that it is natural for women to give birth to babies (Eriksson et al., 2006; Fisher et al., 2006; Ramvi & Tangerud, 2011; Roosevelt & Kane Low, 2016). Women were constantly thinking about future childbirth; they experienced difficulty concentrating (Eriksson et al., 2006; Ramvi & Tangerud, 2011), nightmares about childbirth (Eriksson et al., 2006; Nilsson & Lundgren, 2009; Ramvi & Tangerud, 2011) and they chose to have fewer children than they really desired (Nilsson et al., 2012). Fears for the child’s health and well-being (Ramvi & Tangerud, 2011; Ryding et al., 1998) are fears that all women have, and are not, in our view, contained within the definition of fear of childbirth and fear after childbirth. Other international literature concurs with our results, Matsubara (Matsubara, 2018) and an interview study by Hall (Hall, 2016) with 30 first-time mothers, pre- and post-birth, found there was a pressure on some women to give birth vaginally without pain medication. The women described fears of bodily damage and feelings of awe and pride in the body’s ability to deliver a baby. Hall (Hall, 2016) illustrates the current social pressure on women to adhere to an unspoken norm regarding womanhood and the need to understand women’s response to their bodies during pregnancy and after childbirth, and their feelings about themselves as women (Hall, 2016). The effects of mothers’ fears of childbirth on their babies has not been studied extensively but it is well documented that when mothers are stressed (Kingston, McDonald, Austin, & Tough, 2015), or have depression or other mental health issues, their babies have an increased risk of development or behavioural problems and delayed social-emotional competencies (Kingston & Tough, 2014; McDonald, Kehler, & Tough, 2016). In addition, mother-baby attachment is affected by the mother’s psychological state (Koss, Bidzan, Smutek, & Bidzan, 2016).

All women with fear of childbirth require great support and care so that their pregnancy time, when a woman should feel free to enjoy her coming baby, will not be experienced as a time only of survival. Screening for antenatal anxiety can help women who suffer from fear of childbirth to become aware of their condition and seek help in the form of support from health-care personnel (Evans, Morrell, & Spiby, 2017), Marsay, Manderson, & Subramaney. (2018). Although not having FOC (Dencker et al., 2019; Nilsson et al., 2018), women who experience a high-risk pregnancy also have feelings of fear and anxiety related to their medical condition as well as happiness to be a mother (Wilhelm et al., 2015). To provide sufficient support for women with antenatal anxiety student midwives need education (McGookin, Furber, & Smith, 2017). Also, qualified midwives have been found to need more in-depth knowledge of fear of childbirth (de Vries, Stramrood, Sligter, Sluijs, & van Pampus, 2018), as most of them referred the woman to another caregiver, a psychologist.

Women with fear of childbirth have a five-fold increased risk of having a negative birth experience (Elvander, Cnattingius, & Kjerulff, 2013) but midwives’ empathy and spiritual care can play a key role in the creation of a more positive birth experience. A lack of caregiver empathy, compassion or spiritual care can have consequences for women, such as birth trauma and difficulty bonding with their infants (Moloney & Gair, 2015). Adequate pain relief in labour is essential, but women who have an epidural express greater fear after childbirth postnatally than those who have other pharmacological methods of pain relief (Logtenberg et al., 2018). Probably these women need more pain relief and the epidural is not the cause but a part of a difficult birth.

The women in the reviewed studies described fear of childbirth as a sense of not having control over what was going to happen during pregnancy and childbirth (Faisal et al., 2014; Fisher et al., 2006; Nilsson & Lundgren, 2009; Roosevelt & Kane Low, 2016; Wahlbeck et al., 2018). Lack of control made the women feel in the hands of the staff. The women described their fear of losing control over their body and that they might be ashamed of their own behaviour like screaming and crying while given birth (Faisal et al., 2014; Fisher et al., 2006; Roosevelt & Kane Low, 2016). Nulliparous women described their lack of confidence due to their inexperience of ever giving birth before (Fisher et al., 2006; Melender, 2002). They were afraid of being exposed to severe pain, which they were not able to handle. Multiparous felt ignored with no opportunity to participate and they were concerned about caregiver’s lack of respect (Faisal et al., 2014; Fisher et al., 2006). There are several studies describing women’s perceptions of caregiver’s lack of support. A study by Nilsson (Nilsson, 2014) describes that even if midwives were present in the room, they were not able to give enough attention and support and the women felt ignored. One study (Larkin, Begley, & Devane, 2012) demonstrates that midwives play an essential role in facilitating positive experiences. Control is an important component of childbirth experience and when women received information during the labour process, it was considered a help to feel in control (Larkin et al., 2012).

Some women feared that the body could be injured during labour and birth and caused permanent damage, which they had to live with for the rest of their lives (Melender, 2002; Wahlbeck et al., 2018). This feeling is described by women after a perineal injury which had affected their capacity to care for their newborn child and influenced the relationship with their partner (Priddis, Schmied, & Dahlen, 2014). The women in the reviewed studies also expressed concern that the child could be injured during birth causing a permanent disability (Faisal et al., 2014; Fenwick et al., 2010; Fisher et al., 2006; Melender, 2002; Roosevelt & Kane Low, 2016; Ryding et al., 1998; Wahlbeck et al., 2018). Women’s feeling about lack of confidence in their bodies and worries about their behaviour could be decreased if they had continuity of care during pregnancy and childbirth. A study by de Jonge, Stuij, Eijke and Westerman (de Jonge, Stuij, Eijke, & Westerman, 2014) describes how women felt safe if they had the same caregivers during childbirth.

Women’s self-confidence can be strengthened by a positive birth experience, which could lead to a reduction of fear in the future. This is shown in an interview study of women having midwife-led counselling at a special fear clinic (Larsson et al., 2015). The result demonstrates that through information and knowledge given by midwives, women could feel calm, which decreases the uncertainty of the birth. The feeling of empowerment for women in childbirth is associated with a feeling of safety combined with professional support during childbirth (Larsson et al., 2015).

The women in the reviewed studies described how they were worried about losing control and not being able to master their own behaviour and that they later would be ashamed, for example, for having been shouting uncontrollably. The women also feared that they might experience the labour pain as unbearable (Faisal et al., 2014; Fisher et al., 2006). Previous research demonstrated, with the aim of helping midwives to improve women-centred care; that over 50% of women experienced lack of or loss of control, which contributed to their traumatic birth. Also, fear for the baby’s life was perceived as a cause by 50% percent of the women (Hollander et al., 2017). Previous experiences of this meant that the women believed that they or the baby would die in this situation.

The women in the reviewed studies used different strategies when facing the fear and managing their fear of childbirth. They either chose to confront the fear or tried to displace memories of an earlier traumatic delivery (Eriksson et al., 2006; Fisher et al., 2006; Melender, 2002; Nilsson et al., 2012; Salomonsson et al., 2013; Wahlbeck et al., 2018). They tried to get support from their midwife, to talk to her about the fear (Lyberg & Severinsson, 2010; Salomonsson et al., 2013). The women also tried to take control of their body by practising breathing and relaxation techniques to be used during childbirth (Eriksson et al., 2006; Salomonsson et al., 2013). A study by Campbell and Nolan (Campbell & Nolan, 2016) shows that yoga influences women’s ability to manage labour pain and increases women’s self-efficacy for labour and birth. The time spent in the yoga class was a time with other women in a safe environment where they could discuss their fear in a positive way.

Information and support for all women, with an emphasis on trying to increase tolerance for uncertainty, does alleviate fear of childbirth (Sheen & Slade, 2018). To help nulliparous women with fear, single or group psychoeducation sessions or “therapeutic conversations” in pregnancy may help to improve women’s self-efficacy and reduce the number of requests for CSs due to FOC (Striebich, Mattern, & Averle, 2018). Some women in the reviewed studies wanted to have a CS in order to feel safe and have control over the childbirth (Faisal et al., 2014; Fenwick et al., 2010; Ramvi & Tangerud, 2011). As previously mentioned fear of childbirth is an important driving force for women to request a planned CS (D’Alton & Hehir, 2015; OECD, 2017). However, interventions provided by midwives have shown a reduction of CS, and also a preference for vaginal birth in future pregnancies (Fenwick et al., 2015; Larsson et al., 2015; Ryding et al., 2003). Other studies have shown that women who give birth by CS following maternal request, more often suffer from psychiatric illness both before and after the CS (Möller et al., 2017; Olieman et al., 2017). In an interview study with Japanese pregnant nulliparous women (Takegata et al., 2018), all participants denied preferring CS delivery due to the fear. They were worried about postoperative pain who could have a negative influence on them; not being able to take care for the baby. Motherhood is very important among Japanese mothers, so even if they have fear of childbirth and were terrified about the birth, it was more important to be a good mother to take care of the baby after birth, than to have a good experience of labour (Takegata et al., 2018). Also, Israeli women were concerned about negative outcomes for their baby, fearing their baby might be harmed during birth and they preferred more epidural use than CS. Norwegian women preferred CS compared to Israeli women and were more concerned with the physical and emotional expectations of birth (Preis, Benyamini, Eberhard-Gran, & Garthus-Nigel, 2018). In a recent study by van Dinter-Douma et al. (van Dinter-douma, de Vries, Aarts-Greven, Stramrood, & van Pampus, 2019) it was found that 74% of gynaecologists would grant CS on maternal request if fear persisted despite adequate psychological treatment.

Different forms of interventions have shown positive results on women’s fear (Stoll et al., 2018), but there is no consensus on the best recommended treatment. According to the results of this meta-synthesis, it seems important to offer maternity care that can meet the women’s existential issues allowing them to express and integrate their negative, as well as positive, experiences, feelings and expectations (Larsson, 2018). The care ought to reassure women by listening to them and giving them hope, enabling feelings of control and trust, and supporting them in going into the unknown of pregnancy, labour and birth (Crowther & Hall, 2018; Fahy & Hastie, 2008; Gamble & Creedy, 2009; Wulcan & Nilsson, 2019). Additionally, women who have fear after birth (Dencker et al., 2019), need care that enables them to regain trust in maternity care professionals and their willingness to provide them with good woman-focused care that offers the support the individual women require. Care interventions that meet the existential needs of women have been described in studies on midwives counselling of women with fear of childbirth in Australia and Sweden. This counselling includes similar content and strategies described as “building childbirth resilience” (Fenwick et al., 2013; Gamble & Creedy, 2009; Gamble et al., 2015), and “creating a safe place for exploring fear of childbirth” (Wulcan & Nilsson, 2019).

This review was based on 14 studies of good quality and different methods. The studies originated from Australia, Finland, Iran, Norway, Sweden and from the USA but limiting transferability of findings to other countries, for example, the UK. Maternal care and birthing care in each of these countries occurs within different health-care system and economic insurance regimes. Another limitation of the study is that the quality of the studies varied, which might have affected the result. Therefore, this meta-synthesis could complement the individual studies but could not replace them.

## Conclusions

The women lack guaranties, understanding, control, and they fear pain and injuries. When facing the fear, the women try to manage it through different strategies; they seek support and ask for CS as an emergency exit. The women with fear of childbirth are in need of support that can meet their existential issues about being at a point of no return, allowing them to express and integrate their feelings, experiences and expectations during pregnancy, childbirth and after birth. Health-care professionals need to reassure women by listening to them and giving them hope, enabling feelings of control and trust, and supporting them in going into the unknown of pregnancy, labour and birth. The women suffer physically and emotionally when they try to live with experiences of a terrible childbirth, lack understanding from others and have a feeling of not being themselves. Women with fear after birth (FAB), i.e., after an earlier negative birth experience, need support that enables them to regain trust in maternity care professionals and their willingness to provide them with good care that offers the support that individual women require. Women pregnant for the first time require similar support to reassure them that other´s experiences will not happen to them.

## Appendix

**Final Search strategy, 6 April 2018**

**PubMed**

**Table:**

#1	“Qualitative Research"[Mesh] OR “Grounded Theory"[Mesh] OR “Focus Groups"[Mesh] OR “Interviews as Topic"[Mesh] OR “Narration"[Mesh]	104250
#2	narrative*[tiab] OR narration[tiab] OR interview*[tiab] OR “focus group”[tiab] OR “focus groups”[tiab] OR grounded[tiab] OR qualitative[tiab] OR ethnograph*[tiab]	473522
#3	#1 OR #3	495110
#4	((((fear OR anxiety OR “birth trauma” OR PTSD OR stress disorders))) AND ((birth OR childbirth OR “post partum” OR postpartum OR “post natal” OR postnatal OR puerperium OR antenatal OR prenatal OR perinatal OR parturition OR delivery, obstetric OR cesarean section OR Extraction, Obstetrical OR labor, induced))) AND (("Randomized Controlled Trial"[Publication Type] OR “Observational Study"[Publication Type] OR “Clinical Trial"[Publication Type] OR “Randomized Controlled Trials as Topic"[Mesh Terms] OR “Placebos"[Mesh Terms] OR (random* AND trial*[tiab]) OR “randomized"[tiab] OR “randomly"[tiab] OR placebo* OR “Review” OR “meta-analysis” OR questionnaire OR interview OR survey OR cohort study OR focus group))	8838
#5	#3 AND #4	1870
	Filters activated: English, Swedish	1808
	Publication date from 2015/01/01 to 2018/12/31	537

**PsycINFO**

**Table:**

S1	MAINSUBJECT.EXACT.EXPLODE("Interviews”) OR MAINSUBJECT.EXACT("Narratives”) OR MAINSUBJECT.EXACT.EXPLODE("Grounded Theory”) OR MAINSUBJECT.EXACT.EXPLODE("Qualitative Research”)	39999
S2	ti,ab(narrative* OR narration OR interview* OR “focus group” OR “focus groups” OR grounded OR qualitative OR ethnograph*)	439979
S3	1 OR 2	442548
S4	((ti(Childbirth OR Perinatal OR Postnatal OR Primipara OR Birth) AND ti(Anxiety OR Fear OR “birth trauma”)) OR (ab(Childbirth OR Perinatal OR Postnatal OR Primipara OR Birth) AND ab(Anxiety OR Fear OR “birth trauma”)) OR ((SU.EXACT("Labor (Childbirth)”) OR SU.EXACT("Perinatal Period”) OR SU.EXACT("Postnatal Period”) OR SU.EXACT("Primipara”) OR SU.EXACT("Birth”)) AND (SU.EXACT("Anxiety”) OR SU.EXACT("Birth Trauma”) OR SU.EXACT("Fear”))) AND me.exact("Empirical Study” OR “Quantitative Study” OR “Longitudinal Study” OR “Interview” OR “Followup Study” OR “Literature Review” OR “Prospective Study” OR “Qualitative Study” OR “Clinical Case Study” OR “Treatment Outcome/Clinical Trial” OR “Retrospective Study” OR “Systematic Review” OR “Meta Analysis” OR “Focus Group”))	5593
S5	4 AND 3	909
S5	Limit language English	846
S6	Limit 2015-01-01 - 2018-12-31	214

**CINAHL**

**Table:**

#1	Qualitative studies[MH] OR Discourse Analysis[MH] OR Meta Synthesis[MH] OR Thematic analysis[MH] OR Focus Groups[MH] OR Interviews[MH] OR Narratives[MH] OR Self Report[MH]	→ 89 740
#2	TI (narrative* OR narration OR interview* OR “focus group” OR “focus groups” OR grounded OR qualitative OR ethnograph*) OR AB (narrative* OR narration OR interview* OR “focus group” OR “focus groups” OR grounded OR qualitative OR ethnograph*)	→52 954
#3	#1 AND #2	→96 268
#4	(birth OR childbirth OR “post partum” OR postpartum OR “post natal” OR postnatal OR puerperium OR antenatal OR prenatal OR perinatal OR parturition OR delivery, obstetric OR cesarean section OR labor OR Vacuum Extraction, Obstetrical OR Dystocia) AND (fear OR anxiety OR “birth trauma” OR PTSD OR stress disorders)	→1 396
#5	#3 AND #4	→524
#6	Limit English	(fanns inga svenska)
#7	Limit 2015–2018	

**Cochrane**

**Table:**

#1	((fear or anxiety or “birth trauma” or PTSD or stress disorders) and (birth or childbirth or “post partum” or postpartum or “post natal” or postnatal or puerperium or antenatal or prenatal or perinatal or parturition or delivery, obstetric or cesarean section or Extraction, Obstetrical or labor, induced)):ti,ab,kw	1098
#2	narrative* or narration or interview* or “focus group” or “focus groups” or grounded or qualitative or ethnograph*:ti,ab,kw	30455
#3	#1 AND #2	155
#3	Limit 2015–2018	58

**Scopus**

**Table:**

#1	(TITLE-ABS-KEY (birth OR prenatal OR perinatal OR parturition) OR TITLE-ABS-KEY (“delivery, obstetric” OR “cesarean section” OR “extraction, obstetrical”) OR TITLE-ABS-KEY (“labor, induced” OR antenatal OR puerperium OR postnatal OR “post natal”) OR TITLE-ABS-KEY (postpartum OR “post partum” OR childbirth))	→896 885
#2	TITLE-ABS-KEY (fear OR anxiety OR “birth trauma” OR ptsd OR “stress disorders”)	→474 308
#3	(TITLE-ABS-KEY (“Randomized Controlled Trial” OR “Observational Study” OR “Clinical Trial” OR placebo*) OR TITLE-ABS-KEY (random* OR review OR “meta-analysis”) OR TITLE-ABS-KEY (questionnaire OR interview OR survey OR cohort AND study OR “focus group”))	→8 610 503
#4	#1 AND #2 AND #3	→9 421
#5	TITLE-ABS-KEY (narrative* OR narration OR interview* OR “focus group” OR “focus groups” OR grounded OR qualitative OR ethnograph*)	→1 295 507
#6	#4 AND #5	→2 371
#7	Limit English Swedish	→2 228
#8	Limit 2015–2018	→671

(((TITLE-ABS-KEY (birth OR prenatal OR perinatal OR parturition) OR TITLE-ABS-KEY (“delivery, obstetric” OR “cesarean section” OR “extraction, obstetrical”) OR TITLE-ABS-KEY (“labor, induced” OR antenatal OR puerperium OR postnatal OR “post natal”) OR TITLE-ABS-KEY (postpartum OR “post partum” OR childbirth))) AND (TITLE-ABS-KEY (fear OR anxiety OR “birth trauma” OR ptsd OR “stress disorders”)) AND ((TITLE-ABS-KEY (“Randomized Controlled Trial” OR “Observational Study” OR “Clinical Trial” OR placebo*) OR TITLE-ABS-KEY (random* OR review OR “meta-analysis”) OR TITLE-ABS-KEY (questionnaire OR interview OR survey OR cohort AND study OR “focus group”)))) AND (TITLE-ABS-KEY (narrative* OR narration OR interview* OR “focus group” OR “focus groups” OR grounded OR qualitative OR ethnograph*)) AND (LIMIT-TO (LANGUAGE, “English”) OR LIMIT-TO (LANGUAGE, “Swedish”)) AND (LIMIT-TO (PUBYEAR, 2018) OR LIMIT-TO (PUBYEAR, 2017) OR LIMIT-TO (PUBYEAR, 2016) OR LIMIT-TO (PUBYEAR, 2015)).
